# Cone-Beam Computed Tomography (CBCT)-Guided Surgical Guide for Implant Placement Following Failed Endodontic Retreatment and Alveolar Ridge Preservation: A Case Report

**DOI:** 10.7759/cureus.116134

**Published:** 2026-09-12

**Authors:** Keily J Portillo Avila, Zela Murray, Daymariher Orta Pedroza, Indira D Garzon Zamora, Maria V Urbina Chancay

**Affiliations:** 1 Periodontics, Dent Studio LCB, La Ceiba, HND; 2 Dentistry, Western University of Health Sciences, Pomona, USA; 3 Dentistry, Weston DDS, Weston, USA; 4 Dentistry, Clident, Santo Domingo, ECU; 5 Dentistry, Private Practice, Portoviejo, ECU

**Keywords:** alveolar ridge preservation, cbct, computer-guided implant surgery, dental anxiety, dental implant, endodontic retreatment failure, guided implant placement, periapical infection, surgical guide, tooth extraction

## Abstract

Successful implant rehabilitation depends on accurate diagnosis, adequate preservation of the alveolar ridge, and precise implant positioning. Teeth with persistent periapical pathology following failed endodontic treatment often require extraction, making careful treatment planning essential to achieve predictable functional and esthetic outcomes. The use of cone-beam computed tomography (CBCT) and computer-designed surgical guides has improved the accuracy of implant placement while facilitating prosthetically driven treatment.

We present the case of an American Society of Anesthesiologists (ASA) Physical Status I patient with no known drug allergies who presented with persistent pain and a chronic periapical infection associated with the mandibular left first molar after unsuccessful endodontic treatment and retreatment. The patient also reported significant dental anxiety, which was managed through comprehensive education and step-by-step explanation of every stage of treatment. Following initial pharmacological management, the tooth was atraumatically extracted, and alveolar ridge preservation was performed using particulate bovine bone graft and a resorbable collagen membrane. After four months of healing, CBCT was used to evaluate the regenerated ridge and fabricate a computer-guided surgical template. Implant placement was performed using the surgical guide, followed by a six-month osseointegration period. The restorative phase was completed using a digital workflow with an intraoral scan and scan body to fabricate the definitive implant-supported crown.

Clinical and radiographic follow-up demonstrated successful osseointegration, healthy peri-implant tissues, restoration of function, and a satisfactory esthetic outcome. The patient reported complete resolution of symptoms and a positive treatment experience, with anxiety successfully controlled throughout the multidisciplinary treatment process.

This case highlights the value of CBCT-based surgical guides, alveolar ridge preservation, and digital restorative workflows in achieving predictable implant rehabilitation after failed endodontic retreatment. It also emphasizes the importance of patient education and effective communication in reducing dental anxiety and improving treatment acceptance and overall clinical outcomes.

## Introduction

Persistent apical periodontitis following primary root canal treatment or nonsurgical endodontic retreatment remains a significant clinical challenge and is one of the leading causes of tooth loss in adult patients [1]. Clinical decision-making regarding restorative treatment planning requires careful evaluation of the tooth prognosis and patient-related factors [2]. Previous failed endodontic treatment has also been associated with implant failure risk when extraction becomes necessary [3]. When persistent periapical infection is associated with extensive structural compromise or an unfavorable prognosis, tooth extraction followed by implant-supported rehabilitation is often considered a predictable treatment option.

This case report describes the management of a mandibular first molar with persistent periapical infection following failed endodontic retreatment using atraumatic tooth extraction, alveolar ridge preservation, cone beam computed tomography (CBCT)-guided digital treatment planning, computer-guided implant placement, and a fully digital restorative workflow. The report highlights the integration of contemporary digital technologies with patient-centered communication to achieve predictable functional and esthetic outcomes while successfully managing dental anxiety.

## Case presentation

A 24-year-old medically healthy woman, classified as American Society of Anesthesiologists (ASA) Physical Status I according to the ASA Physical Status Classification System [20], with no known drug allergies, presented with persistent pain associated with the mandibular left first molar (tooth #19). The patient reported a history of primary root canal treatment followed by nonsurgical endodontic retreatment; however, the symptoms persisted. The patient also expressed significant dental anxiety related to previous dental experiences and was apprehensive about undergoing additional treatment.

Clinical examination revealed tenderness to percussion and palpation of tooth #19 without evidence of acute swelling or systemic involvement. Cone beam computed tomography (CBCT) demonstrated a persistent periapical radiolucent lesion associated with the previously treated tooth, confirming persistent apical pathology and an unfavorable long-term prognosis (Figure 1).

**Figure 1:**
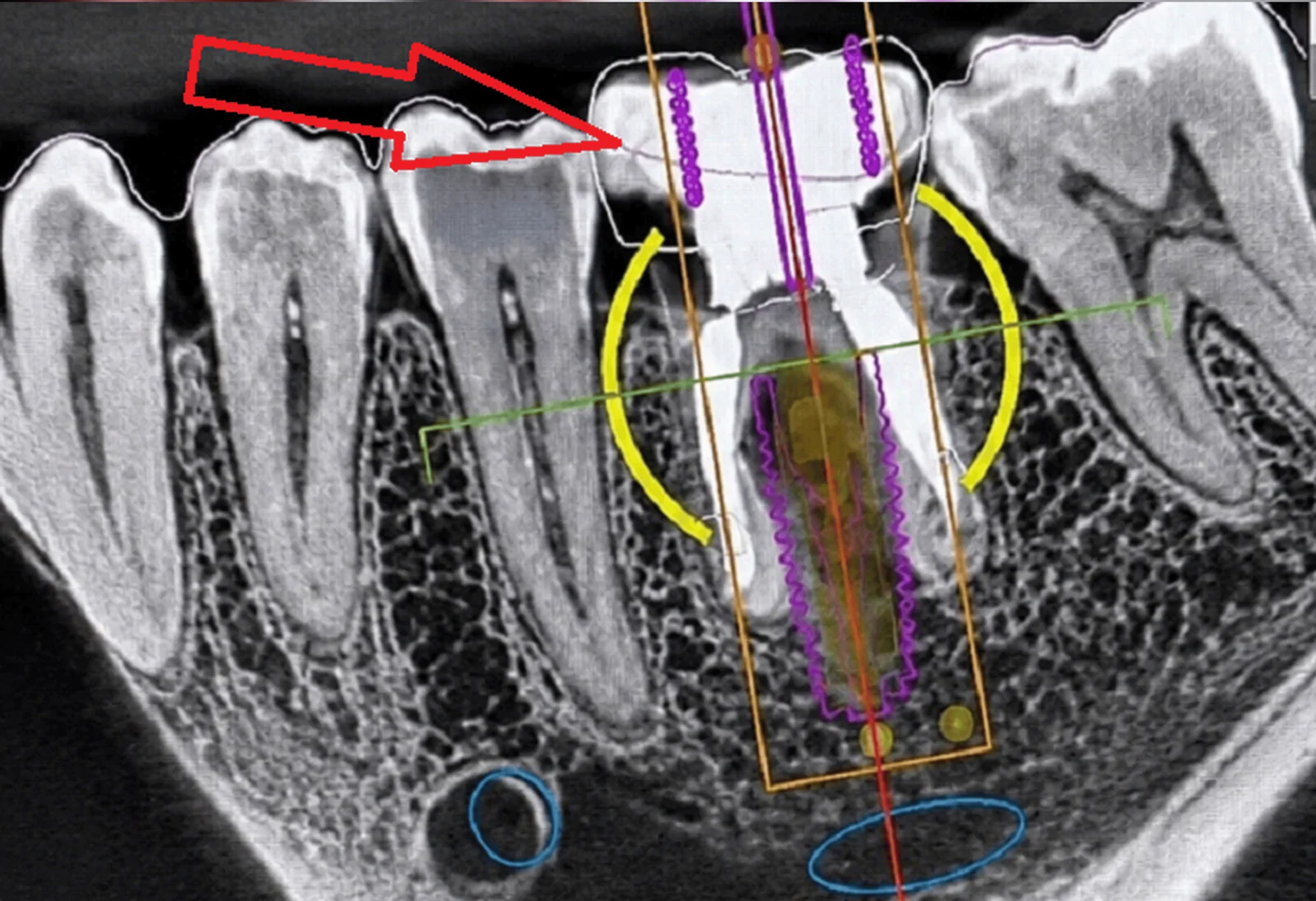
Preoperative CBCT Evaluation of Tooth #19. Preoperative sagittal CBCT view demonstrating persistent periapical radiolucency associated with tooth #19 following failed primary endodontic treatment and nonsurgical retreatment. The red arrow identifies tooth #19, and the yellow marking identifies the associated periapical radiolucency. CBCT: cone beam computed tomography

Initial management included amoxicillin 500 mg orally every eight hours for five days and ibuprofen 600 mg orally every eight hours for three days to control the acute symptoms before surgery. Because of the patient's dental anxiety, extensive counseling was provided before treatment. The diagnosis, treatment alternatives, surgical procedures, expected healing, implant rehabilitation, and restorative phases were discussed in detail using CBCT-based digital treatment planning and a three-dimensional virtual restorative simulation to help the patient visualize the proposed treatment and expected outcome (Figure 2).

**Figure 2:**
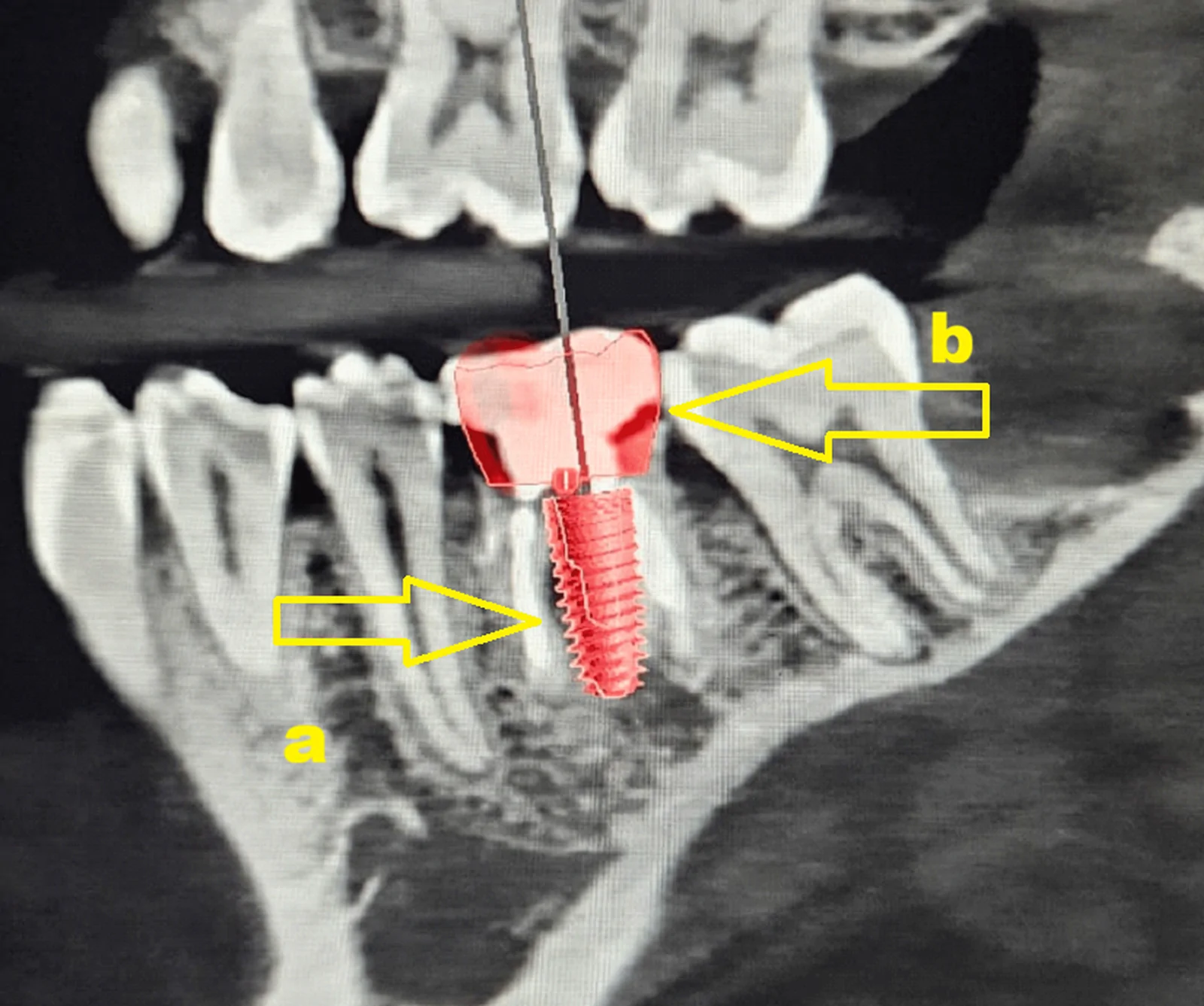
CBCT-Based Digital Implant Planning and Patient Education. CBCT-based digital implant planning performed before surgery. (a) Virtual planning of the implant position within the regenerated alveolar ridge, allowing evaluation of implant angulation, depth, and prosthetically driven placement. (b) Three-dimensional virtual crown used to visualize the planned restorative outcome. The digital treatment plan was also used as an educational tool to explain the procedure, facilitate shared decision-making, and reduce the patient's dental anxiety before implant surgery. CBCT: cone beam computed tomography

This patient-centered approach improved treatment acceptance and significantly reduced anxiety throughout the clinical process.

A preoperative CBCT scan was used to evaluate the alveolar ridge dimensions and surrounding anatomical structures. The digital data were imported into implant planning software to determine the optimal implant diameter, length, angulation, and prosthetically driven position. Virtual implant planning was then completed using CBCT-guided software to verify the three-dimensional implant position in relation to the surrounding anatomical structures (Figure 3).

**Figure 3:**
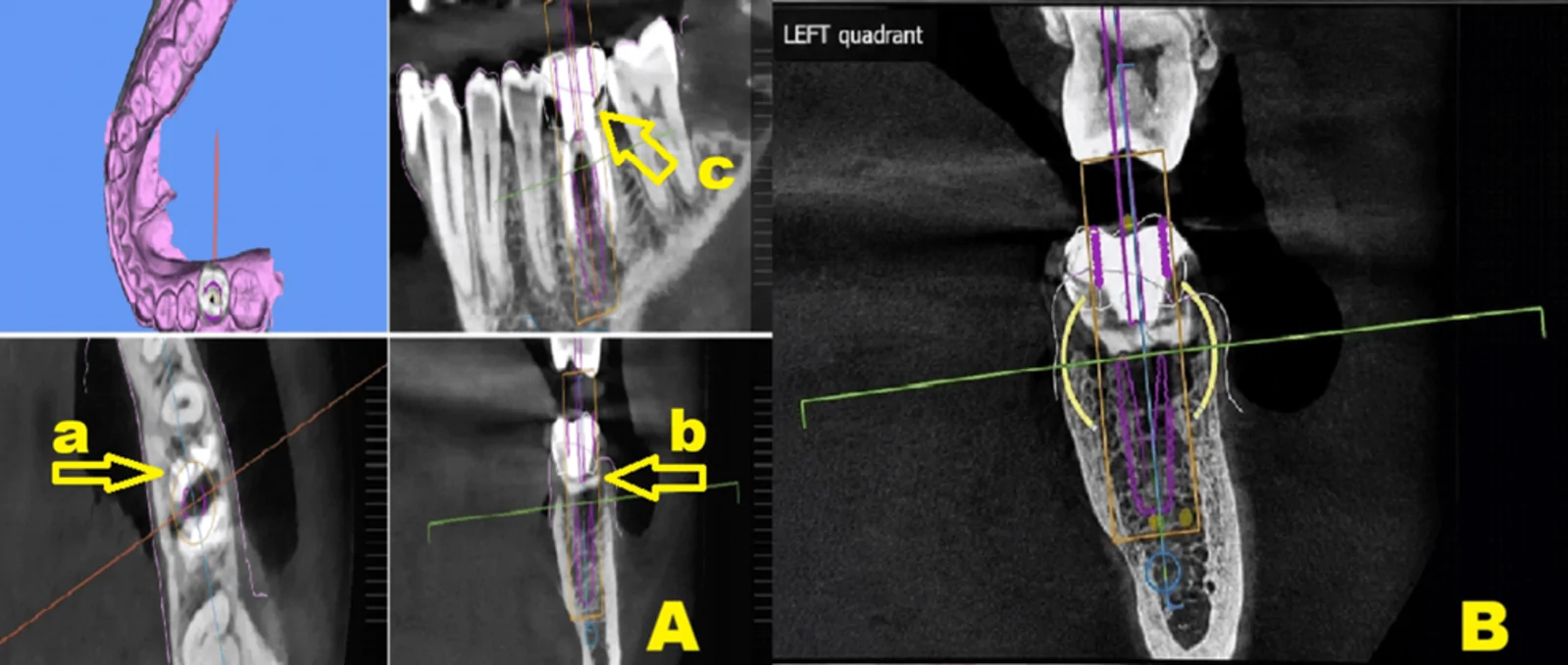
CBCT-Based Surgical Guide Planning. (A) Multiplanar CBCT assessment used for surgical guide planning, including an axial view (a), cross-sectional view (b), panoramic reconstruction (c), and three-dimensional reconstruction. These views were used to evaluate the implant site and determine the planned implant trajectory, angulation, depth, and prosthetically driven position. (B) Enlarged cross-sectional CBCT view demonstrating the virtually planned implant position within the alveolar ridge before guided implant placement. CBCT: cone beam computed tomography

Based on the finalized virtual treatment plan, a three-dimensional printed computer-generated surgical guide was fabricated to accurately transfer the planned implant position, angulation, and depth to the surgical field (Figure 4).

**Figure 4:**
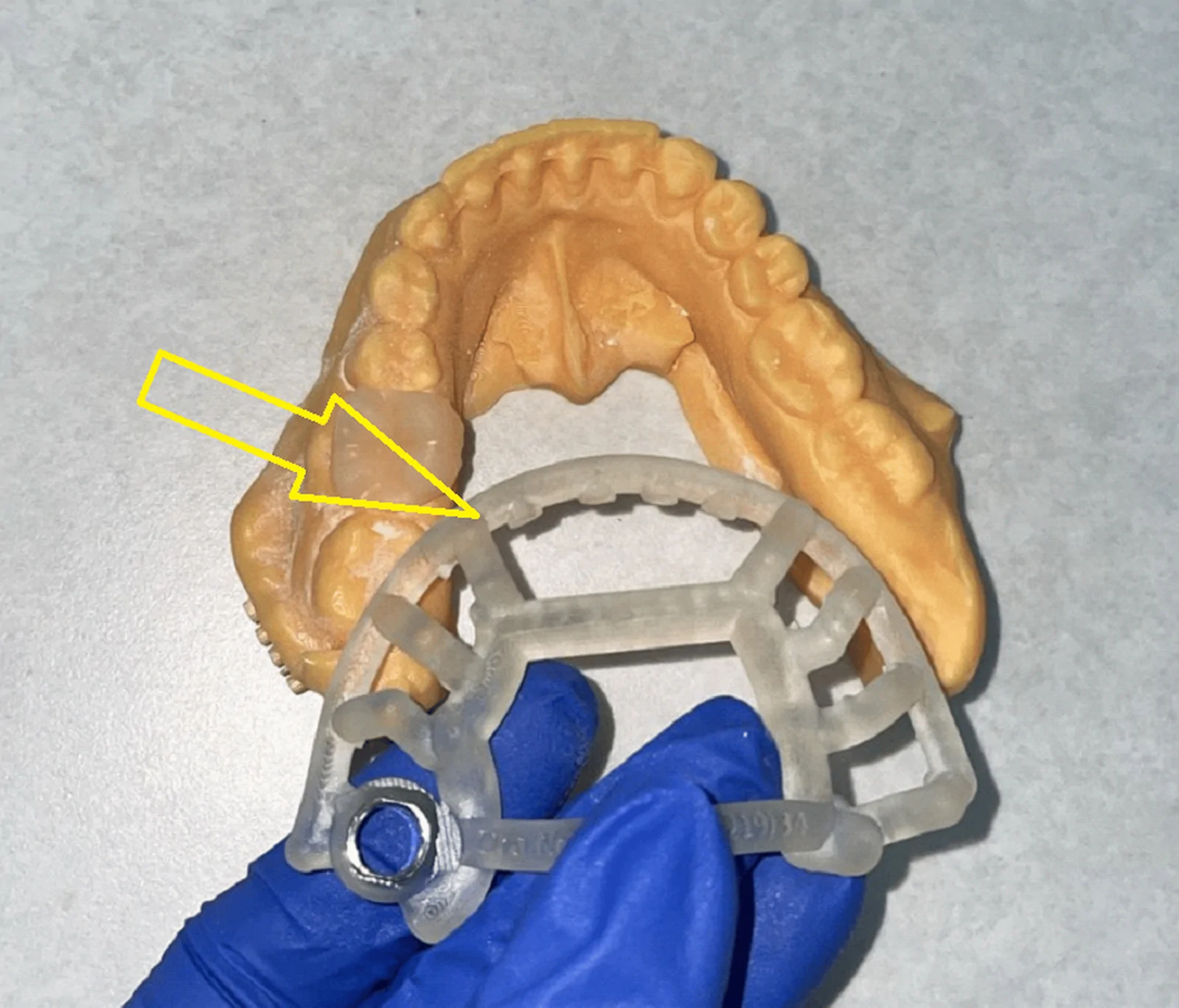
Computer-Generated Surgical Guide for Implant Placement. Three-dimensional printed computer-generated surgical guide fabricated from the digital treatment plan and positioned on the diagnostic cast before surgery. The yellow arrow indicates the metal guide sleeve, which directs the sequential surgical drills and implant placement according to the preplanned angulation, depth, and prosthetically driven position established during the CBCT-based virtual planning. CBCT: cone beam computed tomography

One week after initiation of pharmacological therapy, tooth #19 was atraumatically extracted under local anesthesia. Following complete debridement and irrigation of the extraction socket, alveolar ridge preservation was performed using particulate bovine xenograft covered with a resorbable collagen membrane. Primary wound closure was achieved with interrupted 4-0 nylon sutures (Figure 5).

**Figure 5:**
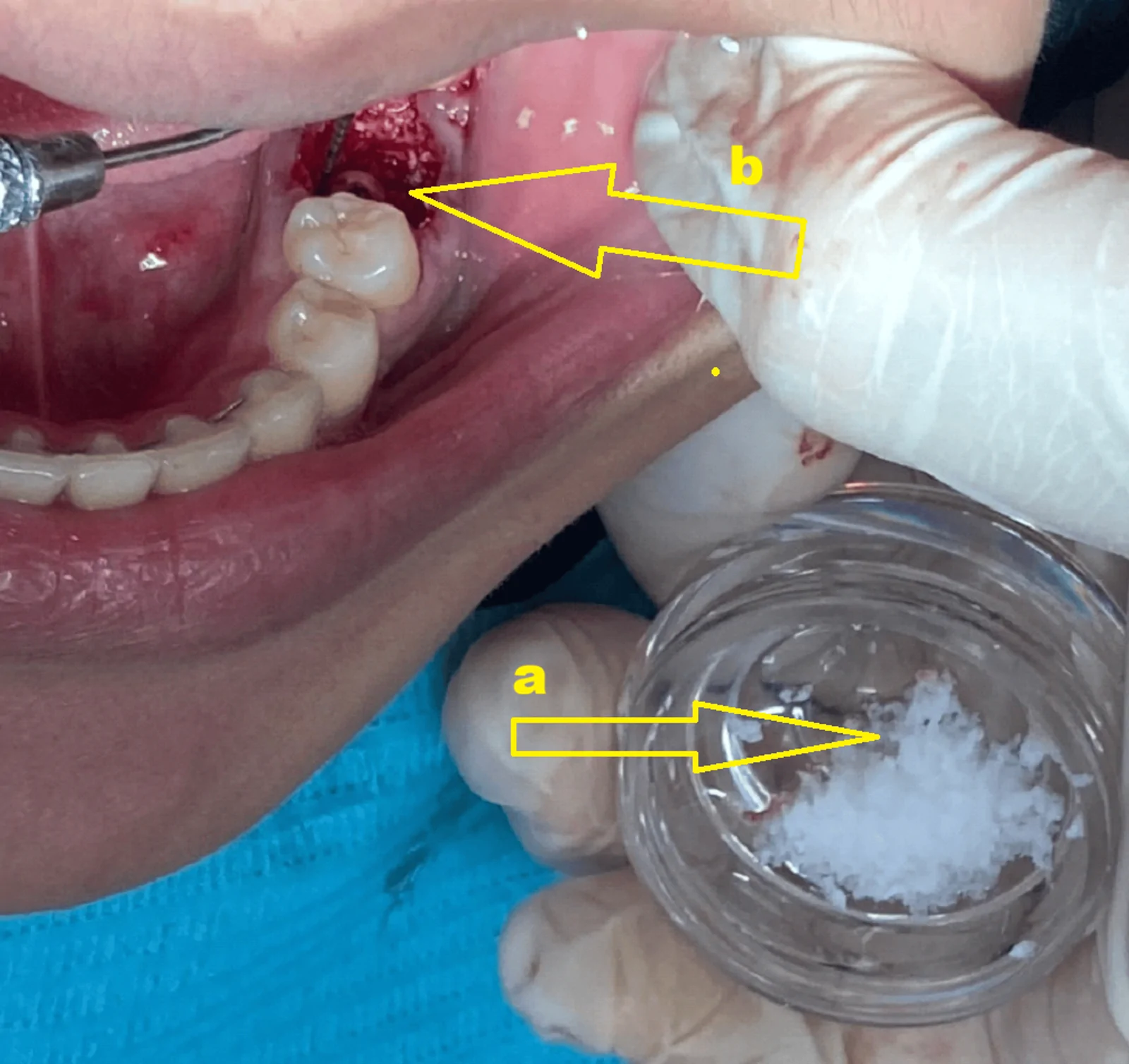
Alveolar Ridge Preservation Following Tooth Extraction. Alveolar ridge preservation performed immediately after atraumatic extraction of tooth #19. (a) Particulate bovine xenograft prepared for grafting of the extraction socket. (b) Placement of the bovine bone graft within the extraction socket to preserve alveolar ridge dimensions before coverage with a resorbable collagen membrane and primary wound closure.

The postoperative course was uneventful, and the sutures were removed 15 days later. After a four-month healing period, clinical and radiographic evaluation demonstrated satisfactory preservation of the ridge dimensions, allowing implant placement in the planned prosthetic position. Using the previously fabricated computer-guided surgical template, the implant was placed with satisfactory primary stability. A six-month healing period was allowed to achieve complete osseointegration.

Following successful osseointegration, the restorative phase was initiated. A scan body was connected to the implant, and a digital intraoral scan was performed (Figure 6).

**Figure 6:**
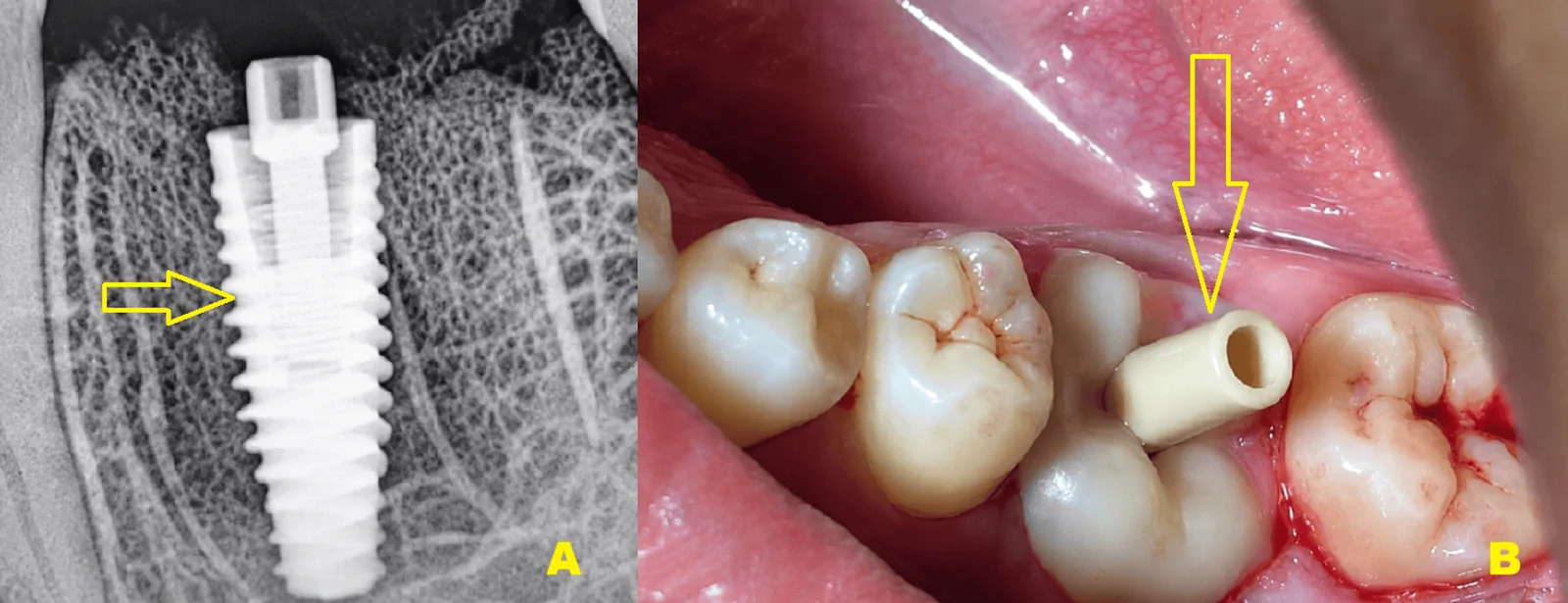
Implant Placement and Digital Restorative Workflow. Implant placement and digital restorative workflow. (A) Periapical radiograph obtained four months after alveolar ridge preservation demonstrating implant placement in the regenerated alveolar ridge with satisfactory positioning. Primary stability was achieved clinically at the time of implant placement. The yellow arrow indicates the dental implant. (B) Clinical photograph obtained after a six-month osseointegration period showing the scan body connected to the implant. The scan body was used during the intraoral digital scan to accurately transfer the three-dimensional implant position to the dental laboratory for CAD/CAM fabrication of the definitive implant-supported crown. The yellow arrow indicates the scan body. CAD/CAM: computer-aided design/computer-aided manufacturing

The digital files were transmitted to the dental laboratory for computer-aided design/computer-aided manufacturing (CAD/CAM) fabrication of the definitive implant-supported crown. Approximately three weeks later, the definitive restoration was delivered and adjusted to establish appropriate occlusal contacts, proximal contacts, and functional occlusion (Figure 7).

**Figure 7:**
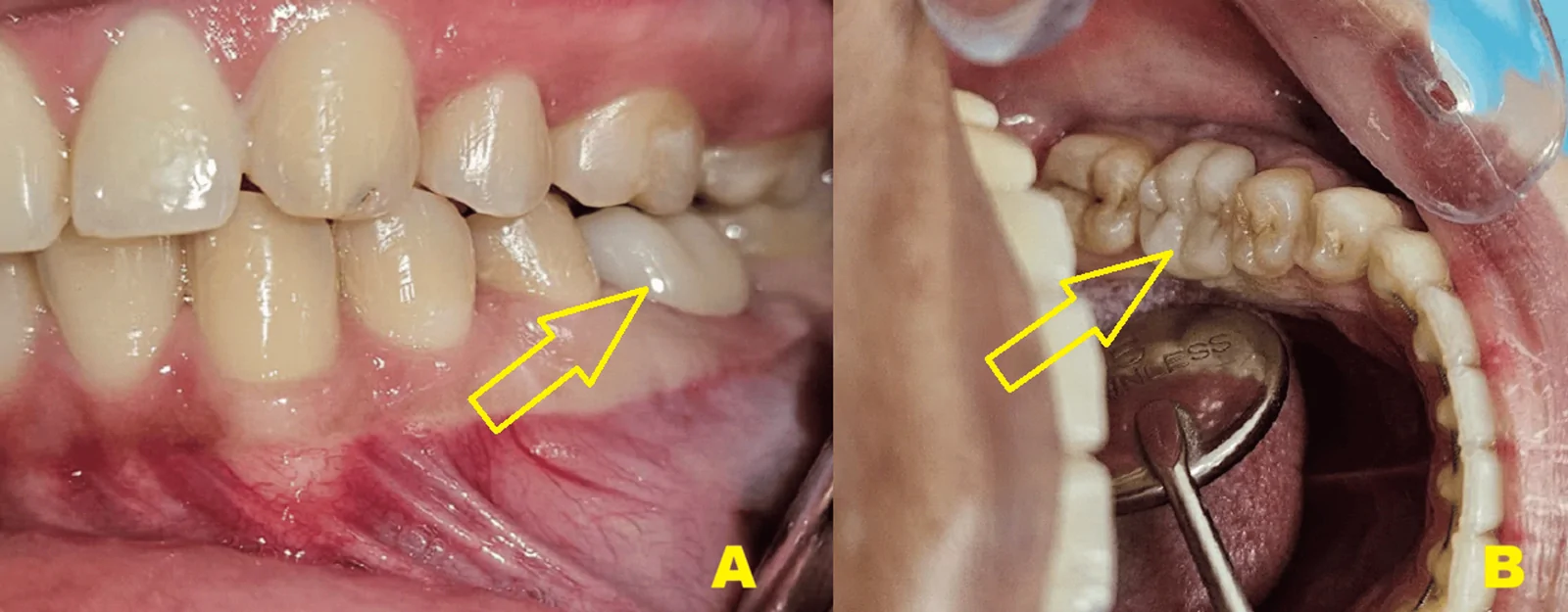
Definitive Implant-Supported Crown. Definitive implant-supported crown after completion of the digital restorative workflow. (A) Buccal view demonstrating the definitive implant-supported crown in harmonious occlusion with the adjacent dentition, exhibiting satisfactory esthetics, proximal contacts, and functional integration. The yellow arrow indicates the definitive implant-supported restoration. (B) Occlusal view demonstrating the anatomical morphology of the definitive crown, appropriate occlusal anatomy, and successful integration with the surrounding dentition. The yellow arrow indicates the definitive implant-supported crown. At the final follow-up, the patient reported complete resolution of symptoms, restoration of masticatory function, and a high level of satisfaction with the treatment outcome.

At the final follow-up examination, the implant demonstrated successful osseointegration with healthy peri-implant soft tissues. The definitive restoration exhibited satisfactory esthetics, proper occlusal integration, and functional stability. The patient reported complete resolution of pain, restoration of masticatory efficiency, and a high level of satisfaction with the treatment.

## Discussion

Persistent apical periodontitis following failed primary root canal treatment or nonsurgical endodontic retreatment represents a common indication for tooth extraction when the long-term prognosis is unfavorable [1]. Clinical decision-making regarding restorative treatment planning should consider the prognosis of the affected tooth [2]. Previous failed endodontic treatment has also been associated with implant-related outcomes when extraction becomes necessary [3]. In these situations, implant-supported rehabilitation offers a predictable treatment option, provided that infection is adequately controlled and sufficient alveolar bone volume is maintained.

Alveolar ridge preservation has become an important component of contemporary implant therapy because it minimizes post-extraction ridge resorption and helps maintain adequate bone dimensions for future implant placement [4]. Systematic reviews have confirmed the benefits of ridge preservation procedures [5]. Randomized clinical trials have demonstrated favorable outcomes using different ridge preservation techniques [6], while clinical recommendations regarding indications and treatment protocols have been well established [7]. CBCT has significantly improved implant treatment planning by providing accurate three-dimensional visualization of the alveolar ridge and surrounding anatomical structures [8]. Contemporary reviews have demonstrated the advantages of computer-guided implant surgery [9], and systematic reviews have confirmed its high level of accuracy and predictability [10].

The guided surgical approach used in the present case contributed to accurate implant placement and facilitated an efficient digital restorative workflow using an intraoral scanner and scan body for fabrication of the definitive implant-supported crown [11]. Complete digital workflows have further improved clinical efficiency, communication with the laboratory, and restorative precision [12]. Long-term tooth survival following endodontic treatment depends on appropriate case selection, infection control, and restorability [13]. Although systemic antibiotics may be indicated in selected situations involving systemic signs of infection, definitive treatment requires elimination of the source of infection [14]. Recent clinical studies have demonstrated favorable outcomes with collagenated xenografts [15], ridge preservation in molar extraction sites [16], and the use of resorbable collagen membranes [17].

Patient-related factors are equally important for successful treatment outcomes. Dental anxiety is frequently associated with avoidance of dental care, increased procedural stress, and reduced treatment acceptance [18]. Effective communication strategies can improve the management of anxious patients in the dental office [19]. In the present case, anxiety management was based on comprehensive patient education, clear communication, and detailed explanation of every phase of treatment. Establishing realistic expectations and involving the patient throughout the treatment process resulted in improved cooperation and a positive overall clinical experience without the need for pharmacologic anxiolysis or conscious sedation.

Although this report describes the successful management of a single patient, its findings should be interpreted within the inherent limitations of a case report. Long-term follow-up and larger prospective clinical studies are necessary to further evaluate the effectiveness and predictability of CBCT-guided implant placement following alveolar ridge preservation in teeth requiring extraction because of failed endodontic retreatment. Nevertheless, this case illustrates how digital treatment planning, guided implant surgery, ridge preservation, and patient-centered communication can be successfully integrated to achieve favorable functional, esthetic, and patient reported outcomes.

## Conclusions

This case demonstrates that CBCT-guided implant planning combined with alveolar ridge preservation can facilitate accurate and predictable implant rehabilitation following failed endodontic retreatment. The integration of three-dimensional imaging, a computer-generated surgical guide, and a digital restorative workflow contributed to successful implant placement, restoration of function, and a favorable esthetic outcome.

This case may also serve as a practical reference for clinicians managing patients with dental anxiety. Explaining each step of treatment, addressing patient concerns, allowing adequate time for questions, and avoiding unnecessary pressure can improve patient confidence, cooperation, and acceptance of care. Although larger clinical studies are needed to confirm these findings, this case highlights how combining digital technology with patient-centered communication can contribute to successful implant rehabilitation and a more positive treatment experience.
